# Wastewater Treatment: Functional Materials and Advanced Technology, 2nd Edition

**DOI:** 10.3390/molecules30224376

**Published:** 2025-11-12

**Authors:** Jingtao Bi

**Affiliations:** Engineering Research Center of Seawater Utilization of Ministry of Education, School of Chemical Engineering and Technology, Hebei University of Technology, Tianjin 300401, China; jingtaob@gmail.com or jingtaob@hebut.edu.cn

Based on the great success of the Special Issue “Wastewater Treatment: Functional Materials and Advanced Technology,” we have launched Volume II in this series. It is striking that more than three years have passed from the publication of the first article of Volume I on 11 May 2022 to the conclusion of Volume II, which comprises a total of 23 papers, including 17 research articles, 5 reviews, and 1 perspective. Together with Volume I, 57 papers have been published in total, an encouraging figure achieved with the support of our colleagues.

From the perspective of wastewater treatment processes, in addition to the adsorption, advanced oxidation, and reduction processes emphasized in Volume I, Volume II has also attracted studies on biocatalysis, dielectric barrier discharge plasma degradation, capacitive deionization, and separation by pervaporation membranes. From the perspective of materials, this Special Issue’s representative systems include metal oxides, bio-based materials, carbon materials, and two-dimensional materials. From the perspective of treatment targets, beyond conventional contaminants, many articles focus on resource recovery from eutrophication-related pollutants, as well as numerous emerging organic pollutants, such as diclofenac and aceclofenac. To facilitate readers’ understanding of the research included in this issue, as in Volume I, I have prepared a keyword word cloud (Figure 1).

In addition to the data analysis of this Special Issue, we also review the completion status of the special aspects proposed in Volume I:

(1) Regarding our goal to “explore the application of relevant materials and technologies in real wastewater and specific application scenarios,” this Special Issue includes a study on electrocoagulation for treating actual drilling wastewater from drilling operation activities in Aksaray Province, and we continue to welcome submissions that address real wastewater and specific application scenarios.

(2) Given that “efficient treatment materials and technologies for emerging pollutants are particularly crucial,” this Special Issue publishes a series of studies on microplastics and other emerging pollutants, addressing this objective well.

(3) Concerning our identification that “intensification technologies are frontier topics worthy of attention,” although this Special Issue includes a number of studies related to electrically assisted processes such as electrocatalysis, this area has not yet been thoroughly approached.

Building on the papers published in Volumes I and II, we are satisfied with the coverage of functional materials and advanced technology for wastewater treatment in these two Special Issues. In view of the many publications on resource recovery in this collection and considering the close relationship between water treatment and resource utilization [1,2], I have decided to continue collaborating with *Molecules* to launch a new Special Issue, “Sustainable Chemical Approaches for Wastewater Treatment and Resource Utilization,” together with Dr. Panpan Zhang of Hebei University of Technology (Available online: https://www.mdpi.com/journal/molecules/special_issues/4353OI640X, accessed on 30 October 2025), and we welcome the attention of the community. However, this also means that the series “Wastewater Treatment: Functional Materials and Advanced Technology” has now come to a close, and I extend my best wishes to every Guest Editor, journal staff member, and author who has worked with us.

## Figures and Tables

**Figure 1:**
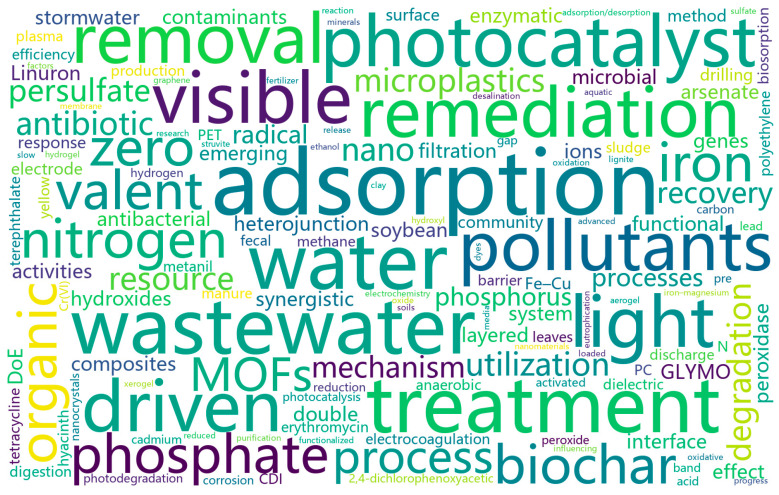
Keyword word cloud prepared for the Special Issue.
